# Exposure to the Four First-Line Anti-Tuberculosis Drugs and Treatment Outcomes: A Target Trial Emulation Study in Ghana

**DOI:** 10.21203/rs.3.rs-9255541/v1

**Published:** 2026-04-10

**Authors:** Michael Opoku-Mireku, Awewura Kwara, Margaret Y. M. Lartey, Charles A. Peloquin, Divine Yao Aseye Amenuke, Kwasi Adjepong Twum, Priscilla A. Nortey, Alexander Ansah Manu, Kwadwo Ansah Koram

**Affiliations:** University of Ghana; University of Florida College of Medicine; Korle Bu Teaching Hospital; University of Florida; Komfo Anokye Teaching Hospital; Komfo Anokye Teaching Hospital; University of Ghana; University of Ghana; University of Ghana

**Keywords:** Tuberculosis, Pharmacokinetics, Treatment failure, Target trial emulation, Subtherapeutic concentrations, Ghana

## Abstract

**Background:**

Tuberculosis (TB) treatment outcomes in sub-Saharan Africa remain suboptimal despite high adherence to first-line therapy. Variability in drug pharmacokinetics, resulting in subtherapeutic plasma concentrations, may contribute to treatment failure and the development of resistance. This study estimated the causal effect of subtherapeutic plasma concentrations of first-line anti-TB drugs on treatment failure or death among individuals with drug-susceptible pulmonary TB in Ghana.

**Methods:**

We conducted a prospective cohort study of 164 adults receiving standard WHO weight-band dosing at five Ghanaian hospitals. Peak plasma concentrations (C_max_) of rifampicin, isoniazid, pyrazinamide, and ethambutol were measured at months 1–2 using validated LC-MS/MS. We emulated a target trial comparing two static strategies: (1) therapeutic C_max_ of at least one first-line drug versus (2) subtherapeutic C_max_ of all four drugs. Using the clone-censor-weight approach, we estimated the per-protocol analogue risk difference (RD) and risk ratio (RR) for treatment failure (smear positive at months 5 or 6) or death by month 6. Models were adjusted for baseline covariates using inverse probability of censoring weighting. Sensitivity analyses included inverse probability weighting with regression adjustment, plain inverse probability weighting, and E-values.

**Results:**

Of 164 participants, 120 had complete pharmacokinetic and outcome data; 20.0% (24/120) had subtherapeutic concentrations of all four drugs. The 6-month risk of treatment failure or death was 33.3% under the low-exposure strategy versus 4.2% under the adequate-exposure strategy (crude RD: 29.1 percentage points). In weighted analyses, low drug exposure was associated with a 25.6 percentage-point increase in absolute risk of treatment failure or death (95% CI: 5.7–45.6; p = 0.012) and an 8.6-fold higher relative risk (95% CI: 2.34–31.92; p = 0.001), corresponding to approximately one additional poor outcome for every four patients with subtherapeutic levels. Sensitivity analyses were consistent (ATE: 19.9%, 95% CI: 2.3–37.5). The E-value was 15.5 (lower bound 5.0).

**Conclusions:**

Subtherapeutic exposure to all four first-line drugs was strongly associated with increased risk of treatment failure or death. Preventing multidrug subtherapeutic exposure through therapeutic drug monitoring or optimized dosing warrants randomized evaluation in high-burden settings.

**Trial registration:**

Clinical trial number: not applicable.

## INTRODUCTION

Tuberculosis remains a major public health challenge, with control efforts falling behind global targets. The WHO End TB strategy targets a 50% reduction in the 2015 incidence rate by 2025, however, by 2023 only an 8.3% reduction had been achieved. The mortality rate reduction achieved in 2023 was 23% of the 2015 rate, although the target for 2025 was 75% (1).

Treatment success rates have improved, reaching 88% for drug-susceptible TB. However, the success rate for drug-resistant TB remains substantially lower at 68%. This disparity underscores the urgent need to prevent the emergence and spread of drug resistance as a key strategy in the global fight against tuberculosis. In sub-Saharan Africa, which bears the highest burden of disease, there has not been much improvement in the treatment success rate in recent years (1). For example, Ghana’s TB treatment success rate has stagnated between 84% and 87% since 2012 (2). Although this represents a substantial improvement from the 50% reported in 2000, the lack of further progress in recent years may be due to a complex interplay of factors that warrant urgent investigation.

A plausible consequence of this observation is the increased emergence of resistant strains of *Mycobacterium tuberculosis* in recent years. Whereas globally, the emergence of resistance among previously treated patients is six times higher compared to treatment-naïve patients, it is estimated to be 19 times higher in Ghana (1, 3). The magnitude of the difference in the rate between previously treated and treatment-naïve patients suggests a treatment-related process that facilitates acquired resistance. Although nonadherence is often thought to be primarily responsible, studies by Pasipanodya and colleagues point to the person-to-person variability in the pharmacokinetics of the drugs rather than nonadherence, unless beyond a low threshold (> 60%) (4, 5). A systematic review and meta-analysis has established that there is a high prevalence of low plasma concentrations of the first-line anti-TB medications at standard doses, thereby substantiating the role of pharmacokinetic variability in promoting resistance (6). These observations underscore the importance of pharmacokinetic variability as a potential determinant of treatment outcomes, providing a key rationale for the present study.

Investigations into the relationship between plasma concentrations of the anti-TB medication at standard doses and treatment outcomes are not conclusive. Despite high adherence to the regimen, poor outcomes may still result (4). Despite high rates of low plasma concentrations successful TB treatment outcome are common (7, 8). The challenge to addressing the dilemma emanates from methodological limitations of the available studies. The evidence is derived from a limited number of studies conducted in settings that may not fully represent the broader population, often involving small sample sizes. Published TB pharmacokinetic studies from Ghana and other West African countries have important limitations. Many were not designed to evaluate the relationship between drug exposure and treatment outcomes or were conducted only among selected clinical subgroups (9-11). Several others relied on retrospective clinical data or did not follow the routine programmatic treatment duration (12-14), reducing their generalizability. These limitations are compounded by the fact that potential confounders were rarely measured or adjusted for, as observed by Perumal and team (7). Even in studies that adjusted for measured confounders, the resulting estimates are primarily associative rather than causal, reflecting biological signals that may not be robust for causal inference. Furthermore, most existing studies focus on individual drug concentrations, even though tuberculosis treatment outcomes arise from the combined synergistic or antagonistic effects of the full multi-drug regimen. This further constrains the interpretability of prior evidence.

A valid estimation of the effect of the pharmacokinetics of the drugs on treatment outcomes is essential. Understanding whether, and how much, low drug exposure despite treatment adherence influences poor outcomes could explain much of the remaining gap in cure rates and open a practical new path to improvement. This would ensure patients receive doses optimized to achieve a cure, thereby raising success rates, shortening infectious periods, and reducing the emergence of resistance. As has been suggested, optimization of the first-line treatment regimen holds the greatest potential for reducing TB incidence and mortality(15, 16).

We therefore analyzed data from a prospective cohort study in Ghana to answer the following causal question: Among patients with drug-susceptible pulmonary tuberculosis treated with standard WHO-recommended weight-band dosing, what is the 6-month risk of treatment failure or death under a hypothetical dosing strategy that guarantees therapeutic exposure to at least one first-line drug from treatment initiation, compared with a hypothetical dosing strategy that results in subtherapeutic exposure to all four first-line drugs? To estimate this effect, we used target trial emulation with the clone-censor-weight approach.

## METHODS

### Target Trial Specification and Emulation

We emulated a two-arm randomized controlled trial comparing treatment strategies among patients with drug-susceptible pulmonary tuberculosis who survived and remained on treatment until pharmacokinetic (PK) assessment (month 1–2):

(1) adequate-exposure strategy: therapeutic peak concentrations (C_max_) of at least one of the four first-line drugs. (2) low-exposure strategy: subtherapeutic C_max_ of all four drugs.

Time zero was defined as the date of pharmacokinetic measurement. Follow-up extended from pharmacokinetic assessment to the end of standard therapy. We sought to estimate the population-averaged risk difference (RD) in poor treatment outcomes (failure or death), comparing the adequate exposure strategy with the low exposure strategy, under the assumptions of exchangeability, positivity, and consistency.

The main components of the target trial are summarized in Table 1.

### Exposure and Outcome Definitions

The C_max_ of the drugs were categorized as “subtherapeutic” if they were below the published threshold and “therapeutic” if equal to or above this threshold (17). The thresholds were 8 μg/ml for rifampicin, 3 μg/ml for isoniazid, 20 μg/ml for pyrazinamide, and 2μg/ml for ethambutol. Because a threshold of 35 μg/ml has been used in clinical practice for pyrazinamide (18, 19), proportions based on both thresholds are presented for completeness. However, all primary analyses were conducted using the 20 μg/ml threshold. The primary outcome was treatment response, categorized as “success” for clinical treatment outcomes of cured and treatment completed, or “poor” for treatment failure and death. Clinical outcome categories were defined using the World Health Organization’s standard criteria (20). A patient was considered cured if they had bacteriologically confirmed pulmonary TB at baseline and became smear negative in the final month of treatment, as well as at least one earlier time point. Treatment completion was assigned to patients who finished the full course of therapy without evidence of treatment failure, but for whom bacteriological results in the final month were unavailable or not performed. Treatment failure referred to patients who remained smear positive at month five or later. Patients who died from any cause during treatment were categorized under mortality. Those who interrupted treatment for two or more consecutive months were classified as lost to follow-up. Adherence was defined as the percentage of prescribed doses missed in the month preceding pharmacokinetic sampling. High adherence was categorized as missing fewer than 10% of doses, while low adherence was defined as missing 10% or more.

### Data source, Eligibility, and Follow-up

Data was obtained from a prospective cohort study conducted at five hospitals (Komfo Anokye Teaching Hospital, Kumasi South Hospital, Holy Family Hospital - Techiman, Suntreso Government Hospital, and Tafo Government Hospital) to investigate the risk factors of poor drug-susceptible tuberculosis treatment outcomes. Patients were enrolled consecutively from 1st August 2022 until the predetermined sample size was reached on 23rd August 2023.

Eligible participants were patients aged ≥ 15 years, weighing at least 30 kg, and had rifampicin-susceptible pulmonary tuberculosis confirmed by GeneXpert MTB/RIF. Patients with multidrug-resistant TB, exclusively extrapulmonary TB, or unknown prior treatment history were excluded. Previously treated patients were included only if their last episode had ended in cure or treatment completion (n = 1 out of the analytic cohort of 120 (0.8%). Peak plasma concentrations of rifampicin, isoniazid, pyrazinamide, and ethambutol were measured once between months 1 and 2 of treatment. Participants were followed monthly until the end of the standard 6-month regimen, with treatment outcomes recorded according to national guidelines.

### Sample size and Recruitment

A sample of size 164 was enrolled. This number was based on an assumed 5% rate of treatment failure or death among adequately exposed patients (21), a design effect of 2.0 to account for clustering by facility, 80% power, and a two-sided α of 0.05, with an additional 20% added for missing pharmacokinetic data or loss to follow-up. Recruitment continued until the target was reached: 92 patients from Komfo Anokye Teaching Hospital, 37 from Holy Family Hospital - Techiman, 21 from Kumasi South Hospital, and 7 each from Suntreso and Tafo Government Hospitals.

### Drug Dosing and Administration

All participants received daily fixed-dose combinations of the four first-line anti-TB drugs during the intensive phase (first 2 months) and rifampicin plus isoniazid during the continuation phase (next 4 months), administered by DOTS in line with WHO recommendations (22). Dosing followed standard weight bands: 30–39 kg (300 mg rifampicin, 150 mg isoniazid, 550 mg ethambutol, 800 mg pyrazinamide), 40–54 kg (450 mg rifampicin, 225 mg isoniazid, 825 mg ethambutol, 1,200 mg pyrazinamide), and ≥ 55 kg (600 mg rifampicin, 300 mg isoniazid, 1,100 mg ethambutol, 1,600 mg pyrazinamide) (23).

### Pharmacokinetic Data Collection and Analysis

At month 1 or 2, 4 ml of blood was collected two and four hours after dosing. Participants fasted overnight (≥ 8 hours), took their observed dose, and received a light meal 30 minutes post-dose. Blood samples were transferred into heparinized tubes, centrifuged at 3,000 g for 10 minutes at 4°C, and the resulting plasma stored at − 80°C until analysis. All specimens were subsequently shipped on dry ice to the Infectious Disease Pharmacokinetic Laboratory at the University of Florida for quantification of drug concentrations. Quantification was done using a validated high-performance liquid chromatography–tandem mass spectrometry (LC-MS/MS) assay. The method demonstrated precision with percentage relative standard deviations between 0.2% and 6.6% across high, medium, and low quality-control levels, and accuracy ranging from 99.03% to 99.67% for all four drugs. Measured concentrations ranged from 0.012–14.382 μg/mL for rifampicin, 0.052–5.162 μg/mL for isoniazid, 0.122–54.142 μg/mL for pyrazinamide, and 0.0052–5.892 μg/mL for ethambutol. Maximum plasma concentrations were estimated by maximum likelihood using the 2-hour and 4-hour post-dose samples modeled in Monolix 2023 (24). Concentrations below the lower limits of quantification (0.25 μg/mL for rifampicin, 0.15 μg/mL for isoniazid, 0.50 μg/mL for pyrazinamide, and 0.05μg/ml ethambutol) were excluded from the C_max_ calculation to prevent unreliable model inputs that would bias C_max_ estimates downward.

### Statistical Analysis and Causal Inference

The pharmacokinetic phenotype reflects a final common pathway of diverse upstream factors, including body weight, malabsorption, drug-drug interactions, genetic variability, adherence, among others, many of which cannot be fully measured in routine settings (25, 26). We estimated the per-protocol effect of sustained adequate versus low pharmacokinetic exposure using standardized static-regime estimation with baseline cloning and artificial censoring. Each participant was duplicated at baseline and assigned to one of two static strategies: therapeutic C_max_ of at least one of the four first-line drugs versus subtherapeutic C_max_ of all four drugs. Clones whose assigned strategy was incompatible with the observed month 1–2 pharmacokinetic phenotype were artificially censored at baseline and excluded from follow-up. Compatible clones were retained in the analysis and weighted by the inverse of the estimated probability of compatibility, conditional on baseline covariates (age, sex, weight, HIV coinfection, diabetes comorbidity, current smoking, adherence, alcohol use, dietary diversity, and baseline smear grade). To reduce variance, weights were stabilized by dividing by the marginal probability of compatibility and winsorized at the 1st and 99th percentiles.

This procedure standardizes outcomes to the baseline covariate distribution and estimates the population-averaged risk difference and risk ratio, and adheres to each exposure phenotype, independent of upstream determinants of exposure. Risk differences and risk ratios were obtained from weighted binomial generalised linear models with identity and log links, respectively, with robust variance estimation clustered by participant. The number needed to treat (NNT) was calculated as the reciprocal of the primary risk difference (RD = 25.6%), yielding an NNT of 4 (95% CI: 2 to 18), indicating that shifting 4 patients from the low-exposure phenotype to adequate exposure would prevent one additional case of treatment failure or death.

### Sensitivity Analysis

To assess the robustness of the primary findings, we conducted three sensitivity analyses. First, we used augmented inverse probability weighted regression adjustment (IPWRA) to estimate the average treatment effect (ATE) of the low-exposure phenotype versus adequate exposure, adjusting for baseline covariates (age, sex, weight, HIV co-infection, diabetes comorbidity, current smoking, adherence, alcohol use, dietary diversity, and smear grade) with robust standard errors. This doubly robust approach remains consistent if either the treatment or outcome model is correctly specified. We also examined covariate balance using standardized mean differences before and after weighting. Raw standardized mean differences (SMD) ranged from − 0.90 to + 0.21 (largest imbalance for weight). After weighting, all SMDs were reduced, with most falling within ∣<0.30∣ (maximum absolute SMD = 0.41 for alcohol; range − 0.30 to + 0.41). Variance ratios remained acceptable (0.45–1.62), supporting effective reweighting. The treated group's effective sample size increased to 54.3 (from raw n = 21), while the control group's effective sample size decreased to 53.7 (from raw n = 87), reflecting the reweighting process to achieve balance.

Second, we applied plain inverse probability weighting (IPW) to estimate the ATE, using the same covariates and robust standard errors, with an oversampling strategy to handle potential positivity violations. Weighted balance was again assessed via standardized mean differences.

Third, we calculated E-values to quantify the strength of unmeasured confounding required to nullify the primary risk difference estimate (RD = 25.6%). The E-value assesses how strongly an unmeasured confounder would need to be associated with both the exposure and outcome (on the risk ratio scale) to explain away the observed association, assuming no true effect (27).

All sensitivity analyses were restricted to patients with complete follow-up (i.e., uncensored patients) and utilized Stata’s “teffects” suite with robust variance estimation. Exploratory Firth-penalized Poisson regression models were fitted to examine individual-drug associations under sparse data and potential separation; these were regarded as hypothesis-generating only and were not used for primary causal inference. Results are shown for completeness only in Additional file 3. All analyses were performed in Stata 17.

### Limitations of the estimand

The clone-censor-weight approach identifies population-averaged effects attributable to the observed pharmacokinetic phenotype, independent of its upstream causes. It does not estimate the effects of hypothetical interventions like therapeutic drug monitoring-guided dosing. Key assumptions include no unmeasured confounding of phenotype-outcome and correct trial specification.

## RESULTS

Of the 164 patients enrolled, 17 (10.4%) were lost to follow-up, and 27 (16.5%) did not have complete four-drug pharmacokinetic measurements at months 1–2, leaving 120 patients (73.2%) for the emulated target trial and all primary analyses. Patients excluded because of loss to follow-up or incomplete pharmacokinetic data (n = 44) were broadly similar to the analytic sample (n = 120). The largest standardized mean differences were 0.47 for baseline weight, 0.46 for treatment facility, and 0.39 for dietary diversity score; all other characteristics had standardized differences < 0.25 (Additional file 1). These same variables also had statistically significant p-values. These modest imbalances indicate that selection into the final analytic cohort introduced limited bias.

Table 2 summarizes the baseline characteristics of participants by their pharmacokinetic phenotype. Of the 120 participants, 85 (70.8%) were male, 81 (67.5%) had a high school education, 77 (64.2%) were employed, 65 (54.2%) were unmarried, and 65 (54.2%) sought care at the Komfo Anokye Teaching Hospital. Mean (SD) age was 41.7 years (13.5), median weight was 58.5 kg (IQR: 52.4, 64.0), and median dietary diversity score was 7.0 (IQR: 5.4, 9.6) out of a maximum of 10. While 20.0% (24/120) were HIV co-infected, 7.5% (9/120) had diabetes, and 38.3% (46/120) were on non-TB medications. Thirteen out of 120 (10.8%) missed 10% or more doses within the first two months of treatment initiation.

Table 3 summarizes the distribution of C_max_ for the four first-line anti-TB drugs. Subtherapeutic C_max_ levels were observed in 93.3% of participants for rifampicin, 86.7% for isoniazid, and 53.3% for ethambutol. For pyrazinamide, using the widely reported threshold of 20 μg/ml, 20.0% of participants had subtherapeutic C_max_. However, when the higher clinical practice threshold of 35 μg/ml is applied, this proportion increases markedly to 80.0%. Overall, 3.3% (4/120) of participants had therapeutic concentrations for all drugs, whereas 20.0% (24/120) exhibited subtherapeutic concentrations for all four medicines.

**Figure 1:**
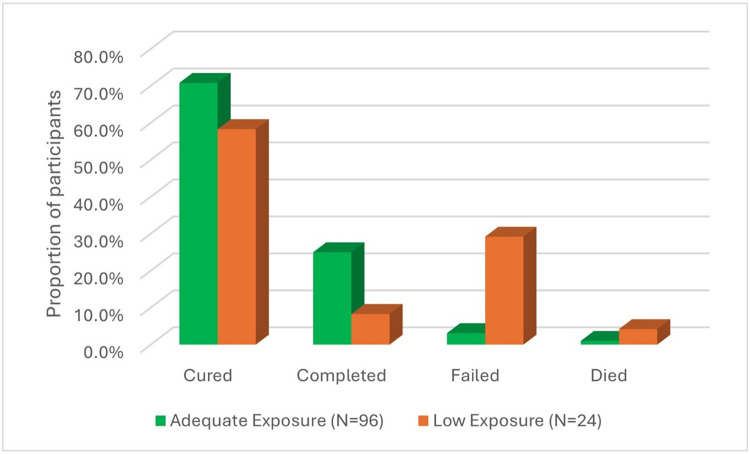
Bar chart showing clinical treatment outcomes by pharmacokinetic phenotype

Clinical outcomes differed between the adequate exposure phenotype and the low exposure phenotype (Fig. 1). Among patients with adequate exposure (n = 96), 95.8% achieved treatment success (70.8% cured; 25.0% completed treatment), and only 4.1% experienced treatment failure or death. In contrast, among those with subtherapeutic concentrations of all four drugs (n = 24), treatment success declined to 66.6% (58.3% cured; 8.3% completed), while poor outcomes increased to 33.4% (29.2% failure; 4.2% death) (p < 0.001). The distribution of baseline covariates by treatment response is presented in Additional file 2.

The results in Table 4 show a strong and consistent link between subtherapeutic C_max_ of all four first-line anti-TB drugs and a much higher risk of treatment failure or death. In the emulated target trial, the 6-month risk of poor treatment response was 33.3% (8 of 24 patients) under the low-exposure strategy compared to 4.2% (4 of 96 patients) under the adequate-exposure strategy. This corresponds to a large and clinically significant causal effect: a crude risk difference of 29.1 percentage points.

In the primary analysis (weighted binomial regression with identity link, accounting for censoring via inverse probability of censoring weights), patients with subtherapeutic levels of all four drugs had an absolute risk increase of 25.6 percentage points (95% CI: 5.7% to 45.6%; p = 0.012) compared to those with adequate levels of at least one drug. This translates to an estimated 3–4 poor outcomes per 100 patients with therapeutic drug levels, rising to approximately 29–50 per 100 among those with low levels of all four drugs. This difference is clearly meaningful. Using a log-linked binomial model (risk ratio scale), the same exposure was associated with an 8.6-fold higher risk of failure or death (RR = 8.64, 95% CI: 2.34–31.92; p = 0.001), meaning patients with low exposure to all four drugs were more than eight times as likely to experience a poor outcome compared to those with better drug levels.

Regarding sensitivity analyses, both IPWRA and plain IPW yielded identical average treatment effects of 19.9 percentage points higher risk (95% CI: 2.3%–37.5%; p = 0.027), after adjustment for age, sex, weight, HIV status, diabetes, smoking, alcohol use, adherence, dietary diversity, and baseline smear grade. The consistency across the two approaches strengthens confidence that the association is not explained by measured confounding or model misspecification (28).

The third sensitivity analysis was the E-value. The point estimate was 15.48, meaning that any unmeasured confounder(s) would need to be associated with both exposure and outcome by a risk ratio of at least 15.5 in both directions to nullify the observed effect. The corresponding E-value for the lower bound of the confidence interval was 4.96, indicating that an unmeasured confounder would still require a substantial risk ratio of approximately 5.0. These high E-values suggest that the association is unlikely to be explained entirely by unmeasured confounding, providing additional support for a causal interpretation of the relationship between low all-four drug exposure and increased risk of poor treatment outcomes.

Overall, these complementary analyses showed that low exposure to all four first-line anti-TB drugs is associated with an elevated risk of treatment failure or death, approximately 20–26 percentage points higher in absolute terms, or more than eight times higher in relative terms.

## DISCUSSION

In this prospective cohort study of adults with drug-susceptible pulmonary tuberculosis in Ghana, subtherapeutic concentrations of all four first-line anti-TB drugs were associated with an increased risk of treatment failure or death. This contrasted with a hypothetical strategy in which therapeutic concentrations of at least one drug were achieved at treatment initiation. The findings were consistent across primary and sensitivity analyses. Moreover, the E-value indicates that an unmeasured confounder would need to be associated with both the exposure and the outcome by risk ratios greater than 15.5 (lower bound 5.0) to nullify the observed effect. Unmeasured confounding greater than 15.5 is implausible since confounders typically exert far smaller effects, but cannot be ruled out (29-32). Even the lower confidence limit (5.7%) represents a significant harm. Our findings suggest that averting the low-exposure phenotype could prevent one poor outcome per 4 patients (NNT=3.9), supporting research into pharmacokinetics-guided interventions.

Our findings are biologically plausible, underscored by the synergistic effect of the drugs in the standard regimen. The clinical efficacy is largely preserved if at least one companion drug achieves adequate exposure, even if the others have low exposure. This may explain the common paradoxical relationship between high overall treatment success rates and low drug concentrations (4,8). However, low exposure to all four drugs disrupts the synergistic bactericidal and sterilizing activity of the regimen, rendering these patients at an elevated risk of failure, death, relapse, and selection of drug-resistant strains (33,34). Ghana’s disproportionately high (19-fold) risk of resistance among previously treated patients compared to treatment-naïve ones likely reflects this phenomenon (1,3).

The high prevalence of sub-therapeutic C_max_ levels for the four drugs, despite high adherence, is consistent with previous studies in sub-Saharan African populations, where malnutrition, drug-drug interactions, and genetic variations affecting drug metabolism are common (7,35). Clinically, this highlights the fact that patients may fail therapy even when they take their medication correctly. This is because standard doses based on weight bands may not achieve the desired therapeutic exposure in all patients. Another potential contributor to subtherapeutic exposure across all four drugs is interindividual variability in drug metabolism. Genetic polymorphisms affecting drug-metabolizing enzymes and transporters, such as *NAT2* polymorphisms governing isoniazid acetylation, transporter variants such as *ABCB1* affecting rifampicin disposition, and hepatic enzymatic pathways involved in pyrazinamide metabolism, are prevalent in African populations and can lead to low plasma concentrations in some individuals (26,36,37). While we did not assess pharmacogenetic markers or metabolite levels, such differences could partly explain the observed phenotype and warrant further investigation in larger cohorts with pharmacokinetic and genetic data.

Our results underscore the need to integrate pharmacokinetic considerations in routine TB care. Implementing therapeutic drug monitoring (TDM) in resource-limited settings like Ghana poses challenges, including the need for advanced laboratory infrastructure and trained personnel. Our study’s use of LC-MS/MS for plasma concentration quantification, conducted at a specialized facility, underscores these logistical barriers. Pragmatic clinical proxies such as early sputum non-conversion, persistent symptoms despite adherence, and markers of severe disease (cavitation, low body weight) can prompt dose intensification (38,39). Alternatively, population pharmacokinetic models that incorporate readily available covariates (weight, HIV status, diabetes, age) can be used to guide empiric dose adjustments (10).

Although LC–MS/MS remains the gold standard for drug quantification due to its high sensitivity and specificity, high-performance liquid chromatography with ultraviolet detection (HPLC-UV) may represent a more affordable alternative in resource-constrained settings (40,41). In addition, dried blood spot (DBS) sampling offers a practical option for therapeutic drug monitoring in remote or decentralized settings, despite being technically more demanding than plasma-based assays (42). These provide opportunities worth exploring to enable the introduction of TDM into routine TB care.

At the population level, the stagnation of treatment success rates in Ghana and similar settings may reflect prevalent low drug exposure rather than programmatic failure alone. If a critical mass of patients systematically achieves subtherapeutic levels of the drugs while on the recommended regimen, even an effective Directly Observed Treatment, Short-course (DOTS) programme cannot fully achieve its expected outcomes. Improving treatment outcomes will require moving beyond adherence-based interventions alone.

Public health programmes need to consider differentiated dosing for at-risk subtherapeutic populations, such as those with comorbidities, or a regimen with optimized doses of one or two of the drugs. In the case of the latter, our study suggested that pyrazinamide and ethambutol concentrations signaled an effect on poor response. It is worth noting that these proposals remain exploratory. This estimand demonstrates that preventing the low-exposure phenotype would substantially reduce poor treatment outcomes. However, it does not establish whether TDM, higher-dose regimens, or any other specific intervention can reliably achieve this phenotype shift in routine clinical practice. Definitive evidence of effectiveness and feasibility will require further randomized studies explicitly designed to test these implementation strategies locally.

To comprehensively address this under-recognized pharmacokinetic barrier to TB elimination, we recommend piloting TDM in high-risk patients at teaching hospitals, accelerating research into higher-dose regimens, and building sustainable local capacity for pharmacokinetic research.

The strengths of this study include its rigorous target trial emulation framework, clearly defining eligibility, time zero, exposure strategies, follow-up, and estimand, reducing common biases like immortal time bias and improving transparency over standard observational analyses. Drug exposure was measured directly via observed C_max_ (not proxies like dose or weight), providing strong biological grounding. Confounding was addressed with stabilized inverse probability weighting and formal balance diagnostics, yielding population-averaged estimates relevant to clinical and policy decisions. Both absolute (risk difference, NNT) and relative (risk ratio) effect measures were reported, enhancing interpretability and relevance. The exposure contrast focused on a severe, biologically extreme phenotype (all four drugs subtherapeutic), making the large observed effect more plausible as a pharmacologic signal. Together, these features strengthen internal validity and support cautious causal interpretation within the study’s assumptions and constraints. Some limitations existed despite these strengths.

Although inverse probability weighting approximated exchangeability, residual confounding remains possible. Unmeasured markers of disease severity (such as cavitary disease, radiographic extent, serum albumin, malabsorption, or inflammatory burden) may influence both exposure and outcomes. While measured covariates achieved acceptable balance post-weighting, causal interpretation depends on the assumption that all important confounders were captured. Exposure was defined at months 1–2, with time zero at pharmacokinetic sampling. The effect, therefore, applies only to patients who survived until sampling. Early deaths or failures before measurement were excluded, limiting generalizability and introducing potential survivor selection bias. A single C_max_ measurement may not fully capture longitudinal exposure. Within-person variability, assay error, or absorption fluctuations could cause misclassification, likely biasing estimates toward the null if nondifferential, though differential misclassification cannot be excluded. Despite stabilized weights and improved balance, the small number of patients with the low-exposure phenotype raises concerns about positivity and weight instability. Extreme weights were truncated, but finite-sample variability may affect precision. Approximately one-quarter of eligible participants were excluded due to missing pharmacokinetic or outcome data. Baseline differences between included and excluded patients suggest potential selection bias, limiting external validity. Finally, the exposure contrast reflects a severe composite phenotype (all four drugs subtherapeutic) rather than drug-specific effects. Findings should not be interpreted as the isolated causal effect of any single agent, but rather as the estimated impact of avoiding profound multidrug subtherapeutic exposure. Despite these limitations, the clearly defined time zero, explicit causal estimand, and robust weighting framework strengthen internal validity relative to conventional regression-based analyses.

## CONCLUSION

Ghana and most sub-Saharan African countries have not been able to acheive TB treatment success above 85–90% despite generally high reported adherence and well-functioning programmes. Our findings suggest that some individuals do not achieve therapeutic concentrations of any of the four first-line drugs on currently recommended doses. Recognizing and correcting this hidden pharmacokinetic shortfall would directly increase the chance of cure for those individuals, shorten infectiousness, and reduce the emergence of drug-resistant strains. These phenotype effects suggest that interventions averting subtherapeutic exposure, such as TDM-guided dosing or empiric high-dose regimens, merit randomized evaluation in high-burden settings like Ghana.

## Supplementary Material

This is a list of supplementary files associated with this preprint. Click to download.

Additionalfile1.docx

Additionalfile2.docx

Additionalfile3.docx

## Figures and Tables

**Table 1:** Specification of the target trial and its emulation using observational pharmacokinetic data

Component	Target Trial (Ideal but infeasible)	Emulation using observational data
Eligibility	Patients aged 15 years or older with a minimum body weight of 30kg, diagnosed with rifampicin-susceptible TB using the GeneXpert MTB/Rif.	Same, but must have survived and remained on treatment until pharmacokinetic sampling (complete-case restriction)
Treatment strategies	Strategy A: Dosing regimen that guarantees a therapeutic C_max_ of at least one of the four first-line drugs from day 0 onward	Strategy A: Observed month 1–2 C_max_ shows therapeutic level for at least one drug (adequate-exposure phenotype)
	B: Dosing regimen that results in subtherapeutic C_max_ of all four first-line drugs from day 0 onward	B: Observed month 1–2 C_max_ subtherapeutic for all four drugs (low-exposure phenotype)
Assignment	Randomization at baseline (day 0)	Clone-censor-weight: each participant cloned and assigned to both strategies; clones censored when their observed exposure violates the assigned strategy
Follow-up start	From day 0 of treatment until month 6, death, treatment failure, or loss to follow-up	Same, with time zero set at pharmacokinetic sampling (month 1–2) and follow-up to month 6
Outcome	Treatment failure or death by month 6	Same
Causal contrast	Intention-to-treat effect	Per-protocol analogue via clone-censor-weighting
Statistical Analysis	Risk difference/ risk ratio	Clone-censor-weight estimation for per-protocol analogue effect of static regimes. Sensitivity analyses included IPWRA, plain IPW, and E-value.
Key assumptions	Randomization ensures exchangeability	Conditional exchangeability, positivity, and consistency given measured covariates (age, sex, weight, HIV, diabetes, smoking, adherence, alcohol, diet, smear grade).

IPW, Inverse Probability Weighting; IPWRA, Inverse Probability Weighted Regression Adjustment

**Table 2:** Baseline characteristics by Pharmacokinetic

Characteristics	Pharmacokinetic Phenotype		
	Total, N = 120	Adequate exposure n = 96	Low exposure n = 24
**Age in years, mean (SD)**	41.7 (13.5)	42.0 (13.9)	40.5 (12.0)
**Weight in kilograms, median (IQR)**	58.5 (52.4, 64.0)	59.0 (53.0, 65.8)	56.0 (49.0, 62.1)
**Dietary diversity Score, median (IQR)**	7.0 (5.4, 9.6)	6.9 (5.4, 9.4)	7.6 (4.9, 9.8)
**Household size, median (IQR)**	4.0 (3.0, 7.0)	4.0 (3.0, 6.5)	4.0 (2.5, 7.5)
**Male sex, n (%)**	85 (70.8)	68 (70.8)	17 (70.8)
**Highest Educational Level, n (%)**			
No formal Education	23 (19.2)	20 (20.8)	3 (12.5)
Primary School	9 (7.5)	9 (9.4)	0 (0.0)
High School	81 (67.5)	62 (64.6)	19 (79.2)
Tertiary	7 (5.8)	5 (5.2)	2 (8.3)
**Employment status, n (%)**			
Unemployed	32 (26.7)	24 (25.0)	8 (33.3)
Employed	77 (64.2)	63 (65.6)	14 (58.3)
Student	8 (6.7)	6 (6.3)	2 (8.3)
Other#	3 (2.5)	3 (3.1)	0 (0.0)
**Not married, n (%)**	65 (54.2)	51 (53.1)	14 (58.3)
**Facility, n (%)**			
Komfo Anokye Teaching Hospital	65 (54.2)	53 (55.2)	12 (50.0)
Suntreso Government Hospital	4 (3.3)	3 (3.1)	1 (4.2)
Kumasi South Hospital	11 (9.2)	9 (9.4)	2 (8.3)
Tafo Government Hospital	4 (3.30	3 (3.1)	1 (4.2)
Holy Family Hospital, Techiman	36 (30.0)	28 (29.2)	8 (33.3)
**Current alcohol use, n (%)**	21 (17.6)	17 (17.9)	4 (16.7)
**Current smoker, n (%)**	8 (6.9)	7 (7.4)	1 (4.5)
**Non-adherent (≥ 10% of doses missed), n (%)**	13 (10.8)	10 (10.4)	3 (12.5)
**Concomitant non-TB drugs, n (%)**	46 (38.3)	38 (39.6)	8 (33.3)
**HIV Co-infected n (%)**	24 (20.0)	21 (21.9)	3 (12.5)
**Diabetes Co-morbidity, n (%)**	9 (7.5)	8 (8.3)	1 (4.2)
**Severe Smear Grade (3+), n (%)**	27 (23.7)	20 (22.2)	7 (29.2)

SD, Standard Deviation; IQR, Interquartile Range

# Includes retirees and apprentices

* Adequate exposure = therapeutic C_max_ of at least one drug

† Low exposure = subtherapeutic C_max_ of all four drugs

**Table 3:** Distribution of peak plasma concentration of anti-TB drugs for participants

Drug	Peak Concentration, μg/ml median (IQR)	Subtherapeutic C_max_ n (%)	Therapeutic C_max_ n (%)
**Rifampicin**	4.1 (2.0, 5.6)	112 (93.3)	8 (6.7)
**Isoniazid**	1.6 (0.7, 2.4)	104 (86.7)	16 (13.3)
**Pyrazinamide (< 20 μg/ml)**	24.9 (12.8, 32.5)	24 (20.0)	96 (80.0)
**Pyrazinamide (< 35 μg/ml)**	24.9 (12.8, 32.5)	96 (80.0)	24 (20.0)
**Ethambutol**	1.9 (1.2, 2.5)	64 (53.3)	56 (46.7)
**All four drugs**	-	24 (20.0)	96 (80.0)

C_max,_ Peak Plasma Concentration; IQR, Interquartile Range; IQR, Interquartile Range

**Table 4:** Risk of Treatment Failure or Death Associated with All Four Subtherapeutic Anti-TB Drugs Exposure

Model Type	Estimand	Effect [95% CI]	P- value	Adjustment
**Primary Analyses (n = 108)**			
Weighted Binomial regression (identity link)	Risk Difference	25.6% [5.7%, 45.6%]	0.012	IPCW-weighted analysis restricted to uncensored cases, with robust standard errors (clustered by participant ID)
Weighted Binomial regression (log link)	Risk Ratio	8.64 [2.34, 31.92]	0.001	IPCW-weighted analysis restricted to uncensored cases, with cluster-robust standard errors by participant ID
**Sensitivity Analyses (n = 108)**			
Inverse Probability Weighting Regression Adjustment	Average Treatment Effect (%)	19.9% [2.3%, 37.5%]	0.027	Robust SE, adjusted for baseline covariates
Inverse Probability Weighting	Average Treatment Effect (%)	19.9% [2.3%, 37.5%]	0.027	Robust SE, oversample strategy, adjusted for baseline covariates
E-value (for primary RD = 25.6%)	E-value	15.48 [Lower bound: 4.96]	-	An unmeasured confounder would need RR ≥15.5 with both exposure & outcome to nullify RD

**Abbreviation:** CI, Confidence Interval;IPCW, Inverse Probability of Censoring Weight; IPW, Inverse Probability Weighting; IPWRA, Inverse Probability Weighting Regression Adjustment; SE, Standard Error

**Notes:** Log-binomial provides a multiplicative RR scale. Covariates were age, sex, weight, HIV, diabetes, smoking, alcohol use, adherence, dietary diversity, and smear grade. All models restricted to uncensored cases (n=108); robust standard errors throughout.

No poor outcomes were observed among patients with therapeutic rifampicin C_max_. However, small numbers precluded formal multivariable estimation (Additional file 2). Low C_max_ was associated with a 7.3-fold (95% CI: 1.89, 28.33) higher risk of treatment failure or death than adequate C_max_. Subtherapeutic C_max_ for pyrazinamide (IRR = 16.7; 95% CI: 4.0–69.4, p < 0.001) and ethambutol (IRR = 4.1; 95% CI: 1.2–13.9, p = 0.024) were each strongly associated with poor treatment response. There wasn't enough evidence of an association between isoniazid C_max_ and treatment response (Additional file 3).
